# SLE: insights from array-based proteomics

**DOI:** 10.1186/ar3978

**Published:** 2012-09-27

**Authors:** T Wu, Y Du, L Davis, C Mohan

**Affiliations:** 1 UT Southwestern Medical Center, Dallas, TX, USA

## 

'Omic'-driven research is exploratory in nature, and seeks to interrogate the entire molecular landscape, with the idea that key pathways or nodes that are aberrant in a disease could be uncovered through a brute-force scan. To date, comprehensive profiling using multiple omics platforms has yielded novel insights on a wide spectrum of diseases, as discussed elsewhere [1].

We recently applied a couple of proteomic and metabolomic approaches to study SLE. In particular, we used planar arrays to uncover novel autoantibodies in SLE, as well as novel serum or urine markers of disease. For the latter, we used planar arrays precoated with antibodies to 274 potential biomarker proteins to interrogate the blood and urine of SLE patients. Thirty of the molecules that were upregulated in SLE sera or urine on these arrays were subsequently validated using independent patient cohorts and orthogonal platforms. In addition to confirming several previously reported increases (including increased serum leptin, osteopontin, OPG, TGF, TNFR-II, and VCAM-1 in SLE), this new study also uncovered several additional proteins to be elevated in SLE.

One example of the novel serum markers being pursued is sTREM-1. sTREM-1 is elevated in the serum of patients with lupus nephritis and within their kidneys. We have also verified that Trem-1 becomes upregulated in mice subjected to anti-GBM nephritis. This molecule may not only be a marker of disease, it may also constitute a novel therapeutic target because Trem-1 blockade curtails nephritis in the anti-GBM experimental nephritis model. Ongoing studies will test the therapeutic potential of this target in spontaneous lupus nephritis. Additional markers uncovered using the arrays will also be discussed.

Omics-based exploratory scans empower us to discern molecules that are differentially expressed in SLE. The challenge ahead is to carefully validate new candidates in order to identify those with the best biomarker or therapeutic potential in SLE.
